# Histopathology in Periprosthetic Joint Infection: When Will the Morphomolecular Diagnosis Be a Reality?

**DOI:** 10.1155/2018/1412701

**Published:** 2018-05-13

**Authors:** G. Bori, M. A. McNally, N. Athanasou

**Affiliations:** ^1^Department of Orthopaedics, Bone and Joint Infection Unit, Hospital Clinic of Barcelona, IDIBAPS, University of Barcelona, Barcelona, Spain; ^2^Nuffield Department of Orthopaedics, Rheumatology and Musculoskeletal Sciences, University of Oxford, Nuffield Orthopaedic Centre, Oxford OX7HE, UK

## Abstract

The presence of a polymorphonuclear neutrophil infiltrate in periprosthetic tissues has been shown to correlate closely with the diagnosis of septic implant failure. The histological criterion considered by the Musculoskeletal Infection Society to be diagnostic of periprosthetic joint infection is “greater than five neutrophils per high-power field in five high-power fields observed from histologic analysis of periprosthetic tissue at ×400 magnification.” Surgeons and pathologists should be aware of the qualifications introduced by different authors during the last years in the histological techniques, samples for histological study, cutoffs used for the diagnosis of infection, and types of patients studied. Recently, immunohistochemistry and histochemistry studies have appeared which suggest that the cutoff point of five polymorphonuclear neutrophils in five high-power fields is too high for the diagnosis of many periprosthetic joint infections. Therefore, morphomolecular techniques could help in the future to achieve a more reliable histological diagnosis of periprosthetic joint infection.

## 1. Introduction

Periprosthetic joint infection (PJI) is one of the most common complications in hip, knee, shoulder, and ankle replacements. For many years, there were no universally accepted criteria for the definitive diagnosis of PJI; each author or scientific society used their own gold standard, which might include clinical, analytical, radiological, microbiological, or histological features. Some authors considered only cultures [1], while others combined histology and cultures [2], and still others added analytical tests [3]. Despite these differences, the histological study of periprosthetic tissue has always been a major component of the attempts to confirm or rule out PJI, and its importance is reflected by its inclusion among the new criteria for PJI infection described by the Musculoskeletal Infection Society (MSIS) in 2011 [4]. Today these criteria have been adopted universally by physicians and surveillance authorities (including the centers for disease control, medical and surgical journals, and the medicolegal community) and by all those involved in the management of PJI [5].

The presence of a polymorphonuclear neutrophil (PMN) infiltrate in periprosthetic tissues has been shown to correlate closely with the diagnosis of septic implant failure. However, the extent of the PMN infiltrate that is required to establish a diagnosis of infection is controversial [6]. The histological criterion considered by the MSIS to be diagnostic of PJI is “greater than five neutrophils per high-power field in five high-power fields observed from histologic analysis of periprosthetic tissue at ×400 magnification” [4]. To many, this definition appears to be oversimplified. The utility of histological diagnosis, in terms of its sensitivity, specificity, and positive and negative predictive values, may vary depending on the technique used, the sample studied, the cutoff point used to define PMN infiltration, and patient- associated factors.

The aim of the present review is to examine the origin of the MSIS' current definition of histological PJI and to consider what morphomolecular studies can add to the histological diagnosis of PJI.

## 2. Histological Techniques

In PJI, two histological techniques have been used: frozen sections for intraoperative histological assessment and paraffin sections for final or postoperative assessment [7]. Classically, both techniques use hematoxylin-eosin staining; both provide information on the likelihood of infection, but their aims are qualitatively different [7]. Intraoperative histology aims to inform the surgeon during the operation whether the prosthesis to be replaced is infected or not. This helps the surgeon to decide whether to implant the definitive prosthesis in an area that is probably infected (a one-stage revision) or to insert a cement spacer with antibiotics before implanting the definitive prosthesis several weeks or months later (a two-stage revision).

The major objective of the definitive postoperative histology is to establish whether the prosthesis was infected. In this regard, it serves as a confirmatory test for infection a posteriori once the new prosthesis has been implanted. Postoperative histology is also useful in diagnosing those cases of PJI which were thought preoperatively, on the basis of clinical and laboratory findings, to be aseptic in nature.

As a result, intraoperative histology is used to guide surgical decisions (i.e., whether or not to implant the definitive prosthesis), and definitive histology, in conjunction with other data such as microbiological results [3], is used to make medical decisions (e.g., whether to administer antibiotics). Another important difference is that although the frozen section diagnosis of septic loosening is based on similar criteria, the morphological identification of neutrophils and their differentiation from other inflammatory elements within periprosthetic tissues is more difficult in frozen sections than in paraffin sections [15]. Some authors report few differences between the results of frozen and paraffin sections, but others have found major discrepancies. Stroh et al. [38] reported a concordance of 97.7% in 304 frozen and permanent sections and the difference did not affect the final outcome of the patients. However, Tohtz et al. [37] reported a 21.8% discrepancy (14 of 64 cases) comparing frozen and paraffin sections. In 12 patients (18.8%), the diagnosis of the frozen sections was ambiguous or unclear, and permanent sections confirmed the diagnosis (the final diagnosis was aseptic loosening in eight patients and septic loosening in four) as the tissue samples were not sufficiently representative for cryohistology. In two patients (3.2%), the diagnosis of the intraoperative frozen section was aseptic loosening and the diagnosis of the permanent sections was septic. Therefore, whenever we evaluate histological results we must be clear whether we are dealing with frozen or paraffin sections, as paraffin section histology avoids or reduces histological technical bias [15].

## 3. Samples for Histological Study

During the revision arthroplasty the surgeon can obtain various samples of periprosthetic tissue for histological analysis. The tissues available are samples of synovium/pseudocapsule, the periprosthetic membrane, and other periprosthetic tissues in which infection is suspected. The literature review (Table 1) shows that the specimens submitted for histological evaluation present considerable variability, and this variability may affect the pathology results. Nevertheless, most authors agree that the best sample for histological study of PJI is the periprosthetic membrane. One study [45] that compared the interface membrane and the pseudocapsule concluded that the interface membrane had a higher sensitivity and predictive values for identifying neutrophils. Specifically, this study found that the proportion of infected patients with positive interface membrane was significantly higher than that among those with positive pseudocapsule (83% versus 42%, *P* = 0.04). A possible reason for these results could be the presence of fibrosis in the pseudocapsule which hindered neutrophil infiltration or that the largest bacterial biofilm is found between implant and bone. In addition, one group [46] recently used membranes (not the pseudocapsule) and have proposed a histopathological consensus classification for a standardized evaluation of periprosthetic tissues. Both these studies [45, 46] support the use of the interface membrane as a reference tissue for histological study.

## 4. Cutoffs Used for the Diagnosis of Infection

The histological criterion used to diagnose whether a prosthesis is infected or not is the presence or absence of PMNs (Table 1). Some authors have also assessed the presence of other cells such as lymphocytes or plasma cells [11, 15, 28]. PMNs are found in infected tissue, but their presence in uninfected tissue is minimal or absent. The results in Table 2 vary because the authors used different gold standards and different patient groups for comparison of the histology tests. The first of these discrepancies may possibly be solved in the future with the introduction of the new definition proposed by the MSIS for periprosthetic infection. The second is more difficult to resolve because it depends on whether all consecutively operated patients are studied or only the ones with a high suspicion of infection [7]. Analysing the histology results from all patients undergoing revision arthroplasty is likely to yield lower specificity and positive predictive values than the results obtained if only patients with a clinical suspicion of infection at the time of surgery are assessed [7].

As with all diagnostic tests, if we raise the histology test's cutoff point for defining infection to ten PMNs, we reduce the sensitivity while increasing the specificity; if we lower it to one PMN, the reverse is the case. The new definition proposed by the MSIS for periprosthetic infection uses five PMNs as cutoff point, because it is the most frequently used worldwide and because several studies have shown that there is no difference between using five or ten PMNs [6, 17, 22]. However, certain microorganisms, especially coagulase-negative staphylococci (CNS) and* P. acnes*, can cause a periprosthetic infection with a PMN infiltration rate below five [11, 23, 35, 42, 47].

## 5. Types of Patients Studied

The type of patient studied may also introduce a major bias in the definition of the sensitivity, specificity, and positive and negative predictive values of histology tests. This is due to the difference in incidence of low-grade infection (CNS and* P. acnes*) or virulent infection. Most authors have tried to assess the true value of this test using the postoperative diagnosis, that is, after the definitive diagnosis of the replacement as septic or aseptic has been established. However, one author assessed the value of the histology test based on the preoperative diagnosis, the suspicion of loosening (either septic or aseptic), or whether it was the time of reimplantation of a definitive prosthesis [23, 28, 35]. This is an interesting strategy, since the distribution of microorganisms responsible for the infection differs in each group [47–49] and this may be the cause of the discrepancies in the test results. When we find patients with a preoperative suspicion of aseptic loosening, only a small number (about 10%) of those with positive cultures are definitely infected, with the microorganisms most commonly responsible for this infection being CNS [23, 47, 48]. Therefore, as Bori et al. [23] reported, histology has low sensitivity in these patients. In a study of 61 replacements with a preoperative suspicion of aseptic loosening, the cultures were positive in 12 cases and CNS were the most common microorganisms (11 cases). Only in six out of 12 cases (50%) did the histology reveal more than five polymorphonuclear leukocytes per high-power field. There is a danger that the high negative predictive value of histology in cases with low suspicion of infection might be used to exclude infection incorrectly.

In patients with a preoperative suspicion of septic loosening, the microorganisms responsible presented a classic distribution of chronic infection with the presence of CNS,* S. aureus*, Gram-negative bacilli, and others; therefore, as many authors have reported [49–51], the histology test is likely to have a high sensitivity since CNS are not the microorganisms with the highest global prevalence. In a study [35] of 38 replacements with a preoperative suspicion of septic loosening (in which CNS were the etiology in 13 cases, Gram-negative bacilli in eight,* Staphylococcus aureus* in seven,* Candida *sp. in two,* Peptococcus *sp. in two,* Enterococcus *sp. in one, and* S. pneumoniae* in one, and no clearly identifiable microorganism was responsible in four), the histology tests were positive in all except two of the 13 caused by CNS.

One interesting group is those recently operated patients who have a cement spacer and require the placement of the definitive prosthesis. As in the first group, positive cultures in these patients are very likely to be due to a CNS or* P. acnes*. The only two specific studies [16, 28] of this group of patients in the literature both conclude that histology has a low sensitivity. In a study [28] with 21 patients at the time of reimplantation, in which seven had positive cultures (six due to CNS and one to* Candida *sp.), the histology was positive in only two cases (one case caused by CNS and the other by* Candida *sp.). The other study [16] reported that only four patients out of 64 were considered to have a persistent infection on the basis of positive intraoperative cultures or permanent histological sections. Overall, intraoperative analysis of frozen sections at the time of reimplantation after resection arthroplasty had a sensitivity of 25%; only one out of four persistent infections was detected. The study did not describe the organisms responsible for the infection.

Most of these studies were performed with revision arthroplasties of the knee and hip, but recently studies of revision arthroplasties of the shoulder [42] and elbow [40] have shown that histology has low sensitivity. This is due not to the type of prosthesis or joint, but to the fact that most infections in shoulder prostheses are due to* P. acnes* and most infections in elbow prostheses are due to CNS* and P. acnes*. In a study [42] of 45 patients with replacements of a shoulder prosthesis, of whom 30 presented infection,* P. acnes* was the etiology in 18 cases and other microorganisms in 12. The sensitivity was lower for the* P. acnes* group (50%) than for the other infections group (67%).

Finally, there are two groups of patients in which histology produces a high rate of false positives for diagnosis of infection: patients who undergo a prosthetic replacement and have an underlying inflammatory disease (e.g., rheumatoid arthritis) [52] and those receiving a prosthetic replacement for a periprosthetic fracture [11, 41]. The first group of patients have a persistent neutrophil infiltration in the periprosthetic tissues due to the underlying active disease and not due to prosthetic infection. Kataoka et al. [52] studied synovial tissue in 60 joints from rheumatoid arthritis patients at the time of the placement of an arthroplasty and found 10 cases with more than five PMNs per high-power field. They concluded that PMNs in the rheumatoid synovium were a common microscopic finding and that the presence of more than five PMNs per high-power field in the rheumatoid synovium was not necessarily consistent with infection. The second group of patients had an acute neutrophil infiltration in periprosthetic tissues due to the fracture. In a study [41] of 11 patients undergoing replacement due to periprosthetic fracture, Muñoz-Mahamud et al. [41] found only two patients with positive cultures, but histology was positive for infection in six cases; that is, the false positive rate was 66.6%. A possible explanation for these results might be the infiltration of neutrophils into the periprosthetic membrane, proceeding from the inflammation secondary to the fracture and from the blood vessels injured during the fracture. Another group in which PMNs can be identified in periprosthetic tissues with increased frequency is that of failed metal-on-metal hip replacements, although numbers greater than five PMNs per high-power field are seen only in microbiologically confirmed cases of PJI [53].

## 6. Is the Morphomolecular Diagnosis the Future?

As we have seen, all the studies analysed to determine the presence of PJI have used hematoxylin-eosin histological staining and have assessed the presence of a neutrophil polymorph infiltrate in periprosthetic tissues. Sometimes it is difficult to identify neutrophils, even using Feldman et al.'s criteria [10]. The Feldman et al.'s criteria are as follows: First, the tissue had to be pink-tan and not simply white scan, to avoid analysis of dense fibrous tissue or fibrin. Second, at least two specific tissue samples were used in order to minimize the risk of sampling error. Third, the five most cellular areas in the tissue sample were chosen for evaluation. Fourth, all polymorphonuclear leukocytes had to have defined cytoplasmic borders to be included. Debris that appeared to be the result of nuclear fragmentation was excluded, as it could not be categorized definitively as a polymorphonuclear leukocyte. Fifth, five separate fields were evaluated under high-power magnification (forty times) and the histology was considered positive for infection if there were more than five polymorphonuclear leukocytes per high-power field in at least five separate microscopic fields. A possible strategy to favor the development of a histological morphomolecular diagnosis would be to stain or identify the presence or absence of PMNs, using the molecular markers that they contain. Two authors [36, 44] have applied this approach in recent clinical studies, though using different strategies. In 2009, Morawietz et al. [36] used immunohistochemistry (CD15), and in 2015, Kashima et al. used histochemistry alone [44]. Morawietz et al. [36] reached the conclusion that 23 PMNs in 10 HPF (visual field diameter 0.625 mm) was the cutoff point to differentiate infected from noninfected tissues (with tissues containing more than 23 PMNs being infected). In this study the authors used CD15 immunohistochemistry to identify PMNs, as follows: The antigen was retrieved with Tris buffer (Target Retrieval Solution High pH; DAKO Cytomation, Glostrup, Denmark) in a pressure cooker for 5 min. Endogenous peroxidase was blocked with 3% peroxide for 10 min. The primary antibody (monoclonal mouse antihuman CD15, clone C3D-1; Dako) was incubated for 30 min at a 1 : 50 dilution. The antibody was visualized with the Labelled Streptavidin–Biotin+ system (Dako) following the manufacturer's instructions. In this way, in contrast to previous clinical studies, identification of PMN was not based on cell morphology alone, but on immunohistochemistry as well. Ideally, PMNs can be identified by their small, lobulated nuclei and their narrow cytoplasmic rim. However, the prosthetic wear-particles or bone fragments, which occur frequently in periprosthetic membranes, make precise microsectioning of these tissues difficult and may lead to artefacts or rather thick sections, complicating the precise identification of PMN. Quantification was therefore performed using CD15 immunohistochemistry for the identification of PMN. The authors [36] concluded that immunostaining obtains more accurate counting of PMN than hematoxylin and eosin staining and PAS staining analysed also in the same study.

**Figure 1:**
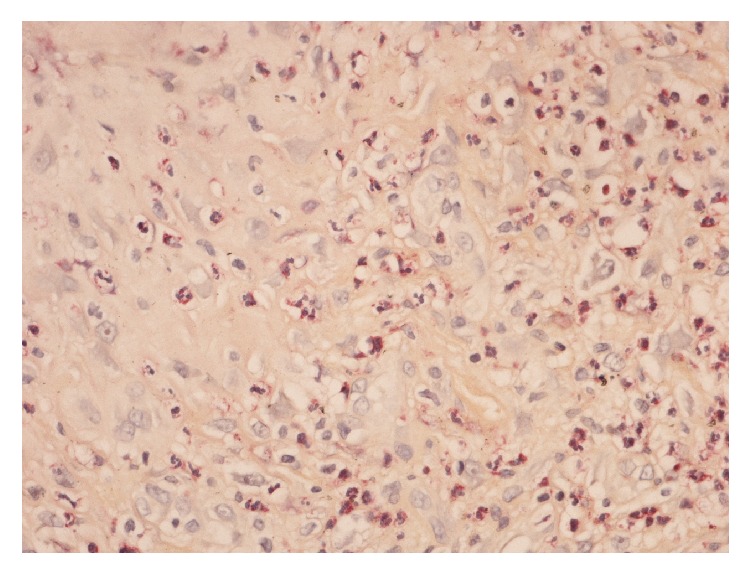
Heavily inflamed granulation tissue in which there are numerous neutrophil polymorphs (>5 per high-power fields) with chloroacetate esterase staining.

**Figure 2:**
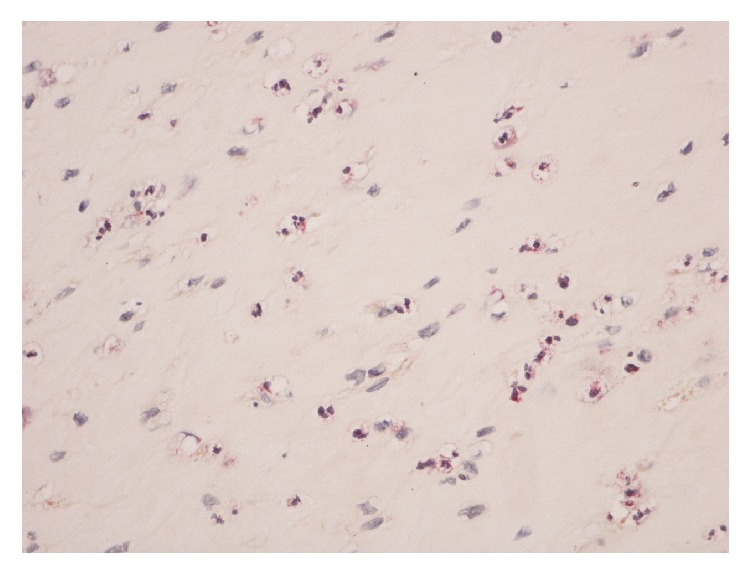
Frozen section of inflammatory tissue showing chloroacetate esterase staining + neutrophil polymorphs (>5 per high-power fields).

**Figure 3:**
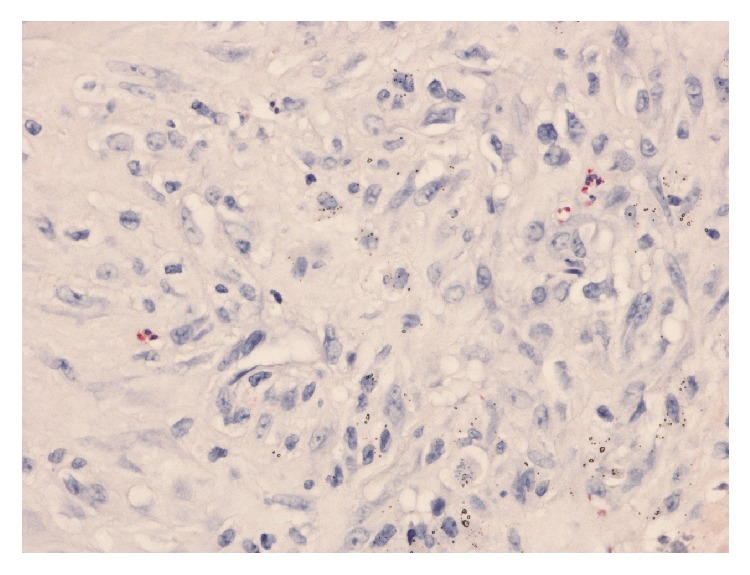
An area of capsular tissue showing chloroacetate esterase staining in which there are fewer than 5 neutrophil polymorphs per high-power field.

Kashima et al. [44] reported that the histological criterion of more than two PMNs per HPF showed increased sensitivity and accuracy for the diagnosis of septic loosening. In that study [44] the authors used chloroacetate esterase (CAE) enzyme histochemistry to identify PMNs, applying the following histological technique: Briefly, Naphthol AS-D chloroacetate (5 mg, SIGMA, St. Louis, MO) in N,N-dimethylformamide was gently mixed with Fast Red GBP Salt (SIGMA) in 0.2 M phosphate buffer, pH 6.4 (5 mg/50 mL). The solution was filtered and applied to sections in a 50 mL Coplinger for 5 min for frozen sections and for 45 min for formalin-fixed paraffin-embedded sections. Sections were counterstained with Mayer's hematoxylin. CAE enzyme histochemistry has been used for many years in hematopathology to detect granulocytes and to distinguish them from other myeloid series cells. In their study, Kashima et al. [44] established that CAE staining facilitates the identification of PMN in frozen and paraffin sections of periprosthetic tissues in cases of septic loosening of hip and knee arthroplasties, and they also reassessed the number of PMNs correlating with septic or aseptic hip and knee implant failure (Figures 1, 2, and 3).

Morawietz et al. [36] and Kashima et al. [44] came to similar conclusions: 23 PMNs in 10 HPF or two PMN in one HPF are indicative of PJI. Their observations suggest that the histological criterion of more than five neutrophils per HPF, considered diagnostic of infection by the MSIS, is too high [54]. A small difference between these two authors is that they use different methods to count the PMNs identified. Morawietz et al. [36] counted all the immunoreactive (red) cells on the CD15-stained slides, regardless of their morphology. In each HPF, a maximum of 10 PMNs was counted. If more PMNs were present in one HPF, the count was limited to 10 PMNs. Ten HPF were examined in this way, so the maximum count per case was 100 PMNs. Kashima et al. [44] examined at least five (×400) HPF (1.55 mm^2^) in five different areas of each histological section (i.e., 25 HPF) and counted the number of PMNs in these five areas. From this, the average number of PMN per HPF was calculated and the polymorph infiltration score determined as follows: 0 means no polymorphs identified, + means fewer than two polymorphs per HPF (×400), ++ means two to five cells per HPF, and +++ means more than five cells per HPF. The ways used to count the PMNs do not seem to affect the conclusions reached by the two authors. Their results corroborate those of previous studies which stated or inferred that infections due to CNS or* P. acnes* might have a PMN infiltration of fewer than five per HPF.

Another strategy for developing the histological morphomolecular diagnosis in PJI is first to define the molecules that are present in infected periprosthetic tissues and absent in uninfected tissues. Recently, two studies [55, 56] have sought to define biomarkers in the synovial fluid in order to identify PJI, but few have defined biomarkers in solid periprosthetic tissues. Testing 16 biomarkers by immunoassay in synovial fluid, Deirmengian et al. [55] found that five biomarkers, namely, human alpha-defensin 1–3, neutrophil elastase 2, bactericidal/permeability-increasing protein, neutrophil gelatinase-associated lipocalin, and lactoferrin, correctly predicted the MSIS classification of all patients, with 100% sensitivity and specificity for the diagnosis of PJI. Therefore, synovial fluid biomarkers may be a valuable addition to the methods used for the diagnosis of PJI in the future. These biomarkers are all host proteins with direct antimicrobial activity, playing important roles in the innate response for eliminating pathogens. When pathogens are present, these biomarkers become more concentrated in the synovial fluid. The problem is that the biomarkers have not been studied in the tissues where they are produced, only in the synovial fluid. Identifying a local host response to bacteria within the periprosthetic tissues would theoretically provide a sensitive and specific test for PJI without the potential for contamination or failure to culture the infecting organism.

CD15 has been the most important tissue biomarker used in clinical and experimental histological studies to distinguish between septic and aseptic loosening [36, 57]. Tamaki et al. [57] reported that aseptic periprosthetic tissue contained numerous CD68-positive monocytes/macrophages in focal stromal cellular infiltrates and in synovial lining. The tissues were also characterized by well-organized and often dense fibrous connective tissues. PMNs were observed only rarely, although a few scattered CD15+ cells were seen in the synovial lining and sublining layers and in perivascular areas. In septic periprosthetic tissues, stromal fibroblasts and marked cellular infiltration with mononuclear cells were observed, associated with fibrous loose connective tissues and a few neovessels. The infiltrating cells were mostly PMNs, which were stained with CD15. The most important problem is that CD15 is not specific for PMN.

Toll-like receptors (TLR) are other tissue biomarkers that have been studied histopathologically in PJI. Takagi et al. [58] and Lähdeoja et al. [59] reported their presence in loosening. Lähdeoja et al. [59] found that the aseptic synovial membrane (aseptic revision) contained markedly more TLR-positive cells per high-power field than osteoarthritic synovium. TLR proteins 1–9 were stained manually using affinity-purified rabbit anti-human IgG antibodies specific for TLR 1 (0.80 mg/mL), TLR 2 (2.7 mg/mL), TLR 3 (2 mg/mL), TLR 4 (1.3 mg/mL), TLR 5 (0,8 mg/mL), TLR 6 (1 mg/mL), TLR 7 (0.8 mg/mL), TLR 8 (2.7 mg/mL), or TLR 9 (0.5 mg/mL), all from Santa Cruz Biotechnology (Santa Cruz, CA). Therefore it seems that prosthetic loosening enhances expression of inflammatory markers that may be useful for morphomolecular diagnosis.

Subsequent studies have tried to identify the specific TLR associated with infection and sought to distinguish between infected and noninfected tissues histologically. Tamaki et al. [57] reported that samples from aseptic loosening, septic loosening, and osteoarthritic synovium showed immunoreactivity for TLR 2, 4, 5, and 9. Monocyte/macrophage infiltrates with marked immunoreactivity of TLR 2, 4, 5, and 9 were observed in the synovial lining in both the interface and regenerated capsular tissues retrieved from aseptically loosened hip joints. In the septic tissues, immunoreactivity to TLR 2, 4, 5, and 9 was detectable in PMN cell infiltrates and in the few monocyte/macrophage-like cells that were also present. In contrast, in osteoarthritis only modest reactivity to TLR 2, 4, 5, and 9 was seen in the endothelial cells and synovial lining. Deirmengian et al. [55] concluded that an increase in expression of TLR can be found in the synovial-like interfacial membrane in aseptic periprosthetic and septic synovial cases compared to osteoarthritic tissues. These TLR cannot be used to differentiate between aseptic and septic tissue in terms of their quantity; however, if we consider their cell location, TLR 2, 4, 5, and 9 were found in monocyte/macrophages in aseptic replacements and in PMNs in septic replacements. Recently, Cipriano et al. [60] in 2014 demonstrated significant increases in the expression of TLR 1 and 6 in infected compared with noninfected tissue obtained during revision total knee or hip arthroplasty. However, TLR1 expression was more accurate in predicting PJI than TLR6 or TLR10. The drawback of this study is that it was not a histological study; the authors used a real-time PCR in homogenized tissue specimens. Therefore, a histological study with TLR1 is required to confirm these results.

## 7. Conclusion

Despite the large number of studies in this field over the past 40 years, the current histological criterion for PJI stipulated by the MSIS (more than five PMNs in five HPF) remains the one proposed by Mirra et al. [8] in 1976. Surgeons and pathologists should be aware of the qualifications introduced by different authors since then, for instance, the fact that infections due to CNS may have an infiltration of fewer than five PMN or that periprosthetic fractures may give false positive results on histological diagnosis. The histological diagnosis is very important in the assessment of PJI, but many hospitals ignore it. Often there may be no pathologist available to make the diagnosis, or communication between the surgeon and the pathologists is poor. Also, surgeons may not be familiar with the histological techniques (HPF, etc.) or do not know the significance of diagnosis established with frozen section or paraffin section histology. In recent years, immunohistochemistry and histochemistry studies have appeared which suggest that the cutoff point of five PMNs in five HPF is too high for the diagnosis of many PJI. Rather than H-E staining (the classical nonspecific staining), these studies use more specific staining for PMN, such as CD15 and CAE. These developments suggest that we should identify the most cost-effective techniques to mark PMN as specifically as possible, so as to be able to identify and count them and make an accurate diagnosis of PJI. Morphomolecular techniques could help to achieve a more reliable histological diagnosis of PJI.

## Figures and Tables

**Table 1:** Summary of the main articles with the type of specimens used for the histological study and the histological criteria for interpretation of histology as diagnostic of infection.

Reference	Specimen	Criteria
Mirra et al. (1976) [8]	Synovial and capsular tissues	≥5 polymorphonuclear leukocytes per HPF in ≥5 HPF (500x)

Fehring and McAlister (1994) [9]	Joint pseudocapsule, interface membrane, and any area that appeared suspicious for possible infection	Evidence of acute inflammation (no quantification)

Feldman et al. (1995) [10]	Joint pseudocapsule and interface membrane	≥5 polymorphonuclear leukocytes per HPF in ≥5 HPF (400x)

Athanasou et al. (1995) [11]	Joint pseudocapsule and interface membrane	≥1 polymorphonuclear leukocyte per HPF on average in at least 10 HPF (400x)^*∗*^

Lonner et al. (1996) [12]	Joint pseudocapsule, interface membrane, and any area that appeared suspicious for possible infection	≥5 and ≥10 polymorphonuclear leukocytes per HPF in ≥5 HPF (400x)

Pace et al. (1997) [13]	Joint pseudocapsule and interface membrane	≥5 polymorphonuclear leukocytes per HPF on multiple (three) HPF (600x)

Abdul-Karim et al. (1998) [14]	Interface membrane (aseptic suspicion). Interface membrane, synovial tissue, and unusually discolored tissue (septic suspicion)	≥5 polymorphonuclear leukocytes per HPF in ≥5 HPF (400x)

Spangehl et al. (1999) [3]	Synovial surface	≥5 polymorphonuclear leukocytes in any single HPF (400x)

Pandey et al. (1999) [15]	Joint pseudocapsule and interface membrane	≥1 polymorphonuclear leukocyte per HPF on average in at least 10 HPF (400x)^*∗*^

Pons et al. (1999) [2]	Synovial surface	≥5 polymorphonuclear leukocytes per HPF in ≥5 HPF (400x)

Della Valle et al. (1999) [16]	Joint pseudocapsule, granulation tissue, and any area that appeared suspicious for possible infection	-

Banit et al. (2002) [17]	Joint pseudocapsule and any area that appeared suspicious for possible infection	≥10 polymorphonuclear leukocytes per HPF in ≥5 HPF (400x)

Musso et al. (2003) [18]	Joint pseudocapsule, interface membrane, and any area that appeared suspicious for possible infection	≥5 polymorphonuclear leukocytes per HPF in ≥5 HPF (400x)

Malhorta and Morgan (2004) [19]	Joint pseudocapsule	≥5 polymorphonuclear leukocytes per HPF in most areas (400x)

Ko et al. (2005) [20]	Joint pseudocapsule, interface membrane, and any area that appeared suspicious for possible infection	≥5 polymorphonuclear leukocytes in any single HPF (400x)

Wong et al. (2005) [21]	Synovial surface, joint pseudocapsule, and interface membrane	≥5 and ≥10 polymorphonuclear leukocytes per HPF in ≥5 HPF (400x)

Francés Borrego et al. (2006) [22]	Periprosthetic soft tissue	≥10 polymorphonuclear leukocytes in any single HPF (400x)

Bori et al. (2006) [23]	Joint pseudocapsule, interface membrane, and any area that appeared suspicious for possible infection	≥5 polymorphonuclear leukocytes per HPF in ≥5 HPF (400x)

Morawietz et al. (2006) [24]	Interface membrane	Evidence of acute inflammation (no quantification). Low or high grade.

Nuñez et al. (2007) [25]	Joint pseudocapsule, interface membrane, and any area that appeared suspicious for possible infection	≥5 polymorphonuclear leukocytes per HPF in ≥5 HPF (400x)

Nilsdotter-Augustinsson et al. (2007) [26]	Synovial surface and interface membrane	≥5 polymorphonuclear leukocytes in any single HPF (400x)

Della Valle et al. (2007) [27]	Synovial surface	≥10 polymorphonuclear leukocytes per HPF in ≥5 HPF (400x)

Bori et al. (2007) [28]	Joint pseudocapsule, interface membrane, and any area that appeared suspicious for possible infection	≥5 polymorphonuclear leukocytes per HPF in ≥5 HPF (400x)

Kanner et al. (2008) [29]	Periprosthetic soft tissue	≥5 polymorphonuclear leukocytes per HPF in ≥5 HPF (400x)

Müller et al. (2008) [30]	Interface membrane	Evidence of acute inflammation (no quantification)

Schinsky et al. (2008) [31]	Synovial surface	≥10 polymorphonuclear leukocytes per HPF in ≥5 HPF (400x)

Fink et al. (2008) [32]	Periprosthetic tissue	≥5 polymorphonuclear leukocytes in any single HPF (400x)

Schäfer et al. (2008) [33]	Periprosthetic soft tissue and membrane	≥5 polymorphonuclear leukocytes per HPF in ≥10 HPF (400x)

Savarino et al. (2009) [34]	-	≥1 polymorphonuclear leukocytes in any single HPF (600x)

Bori et al. (2009) [35]	Joint pseudocapsule, interface membrane, and any area that appeared suspicious for possible infection	≥5 polymorphonuclear leukocytes per HPF in ≥5 HPF (400x)

Morawietz et al. (2009) [36]	Interface membrane	≥23 polymorphonuclear leukocytes in ≥10 HPF (400x)^*∗∗*^

Tohtz et al. (2010) [37]	Interface membrane	≥2 polymorphonuclear leukocytes per HPF in at least 10 HPF (400x)

Stroh et al. (2012) [38]	Joint pseudocapsule, synovium, and soft tissue	Mean of greater than 5 polymorphonucleocytes (PMNs) per HPF was the criteria

Miyamae et al. (2013) [39]	Periprosthetic tissue	≥10 polymorphonuclear leukocytes in any single HPF (400x)

Ahmadi et al. (2013) [40]	Periprosthetic tissue	≥5 polymorphonuclear leukocytes in any single HPF (400x)

Muñoz-Mahamud et al. (2013) [41]	Interface membrane	≥5 polymorphonuclear leukocytes per HPF in ≥5 HPF (400x)

Grosso et al. (2014) [42]	Joint pseudocapsule and interface membrane	≥10 polymorphonuclear leukocytes per HPF in ≥5 HPF (400x)

Buttaro et al. (2015) [43]	Joint pseudocapsule, interface membrane, and any other tissue involved according to the surgeon's judgment	≥5 polymorphonuclear leukocytes per HPF in at least 10 HPF (400x)

Kashima et al. (2015) [44]	Joint pseudocapsule and interface membrane	≥2 polymorphonuclear leukocytes per HPF on average in at least 10 HPF (400x)^*∗∗∗*^

^*∗*^≥1 polymorphonuclear leukocyte per HPF on average after examination of at least 10 HPF; ^*∗∗*^≥23 polymorphonuclear leukocytes in ≥10 HPF (400x). In each HPF, a maximum of 10 polymorphonuclear leukocytes were counted. The sum must be between zero and 100; ^*∗∗∗*^≥2 polymorphonuclear leukocytes per HPF on average after examination of at least 10 HPF.

**Table 2:** Sensitivity, specificity, and positive and negative predictive values.

	*N*	Cutoff PMN	*S* (%)	*E* (%)	PPV (%)	NPV (%)
Mirra et al. (1976) [8]	34	5	100	98	-	-
Fehring and McAlister (1994) [9]	107	Total	18	89	-	-
Feldman et al. (1995) [10]	33	5	100	96	-	-
Athanasou et al. (1995) [11]	106	1	90	96	88	98
Lonner et al. (1996) [12]	175	5	84	96	70	98
Lonner et al. (1996) [12]	175	10	84	99	89	98
Pace et al. (1997) [13]	25	5	82	93	90	87
Abdul-Karim et al. (1998) [14]	64	5	43	97	-	-
Spangehl et al. (1999) [3]	202	5	80	94	74	96
Pons et al. (1999) [2]	83	5	100	98	94	100
Della Valle et al. (1999) [16]	64^*∗*^	5	25	98	50	95
Banit et al. (2002) [17]	121	10 (knee and hip)	67	93	67	93
Banit et al. (2002) [17]	55	10 (knee)	100	96	82	100
Banit et al. (2002) [17]	63	10 (hip)	45	92	55	88
Musso et al. (2003) [18]	45	5	50	95	60	92
Ko et al. (2005) [20]	40	5	67	97	86	91
Wong et al. (2005) [21]	40	5	93	77	68	95
Wong et al. (2005) [21]	40	10	86	85	75	92
Francés Borrego et al. (2006) [22]	63	10 (knee)	66	89	81	81
Francés Borrego et al. (2006) [22]	83	10 (hip)	50	100	100	95
Bori et al. (2006) [23]	61	5	50	81	40	86
Nuñez et al. (2007) [25]	136	5	85	87	79	91
Nilsdotter-Augustinsson et al. (2007) [26]	85	5	81	100	100	87
Della Valle et al. (2007) [27]	105	10 (knee)	88	96	91	93
Bori et al. (2007) [28]	21	5	28	100	100	73
Bori et al. (2007) [28]	21	1	71	64	50	81
Kanner et al. (2008) [29]	132	5	29	95	40	92
Müller et al. (2008) [30]	37	Total	94	94	97	86
Schinsky et al. (2008) [31]	201	10 (hip)	73	94	82	90
Fink et al. (2008) [32]	145	5	90	95	88	96
Savarino et al. (2009) [34]	31	1	80	100	100	80
Morawietz et al. (2009) [36]	147	23^*∗*^	73	95	91	84
Tohtz et al. (2010) [37]	52	23^*∗*^	86	100	100	94
Miyamae et al. (2013) [39]	86	10	71	89	42	97
Ahmadi et al. (2013) [40]	227	5 (elbow)	51	93	60	90
Muñoz-Mahamud et al. (2013) [41]	11	5 (fracture)	100	55	33	100
Grosso et al. (2014) [42]	44	5 (shoulder)	57	100	-	-
Grosso et al. (2014) [42]	44	10 (shoulder)	73	100	-	-
Buttaro et al. (2015) [43]	76	5	90	94	87	96
Kashima et al. (2015) [44]	76	2	94	97	-	-
Kashima et al. (2015) [44]	76	5	83	97	-	-

*N*: number of patients, PMN: polymorphonuclear neutrophil, *S*: sensitivity, Sp: specificity, PPV: positive predictive value, NPV: negative predictive value; ^*∗*^≥23 polymorphonuclear leukocytes in ≥10 HPF (400x). In each HPF, a maximum of 10 polymorphonuclear leukocytes were counted. The sum must be between zero and 100.
